# Fifteen years after a ferry disaster: clinical interviews and survivors’ self-assessment of their experience

**DOI:** 10.3402/ejpt.v4i0.20650

**Published:** 2013-10-03

**Authors:** Filip K. Arnberg, Christina M. Hultman, Per-Olof Michel, Tom Lundin

**Affiliations:** 1 Department of Neuroscience, National Centre for Disaster Psychiatry, Uppsala University, Uppsala, Sweden; 2 Department of Medical Epidemiology and Biostatistics, Karolinska Institutet, Stockholm, Sweden

**Keywords:** Survivors, posttraumatic stress disorder, diagnosis, social support, posttraumatic growth, mixed methods

## Abstract

**Background:**

Disasters yield increased rates of psychological disorders decades later. Other consequences, however, have received little attention in the past.

**Objective:**

We aimed to examine diagnostic status and survivors’ views on disaster-related consequences and social support.

**Methods:**

A mixed-methods approach was used with 22 survivors (of 49 eligible) 15 years after a ferry disaster. Data collection included audiotaped interviews with open-ended questions and diagnostic assessment of Axis-I disorders.

**Results:**

The post-disaster incidence was 54% (12/22) for Axis-I disorders, and 45% (10/22) for full or subsyndromal posttraumatic stress disorder. Thematic analysis revealed that survivor perception of the long-term consequences included positive (character change) and negative aspects (being ascribed a survivor identity). Participants’ sought social support for several years, yet many felt hindered by experiential dissimilarity and distress of significant others.

**Conclusions:**

Axis-I disorders were prevalent, but not salient to survivors’ perceptions in the long-term. Post-disaster interventions need to attend to common barriers to support.

A salient consequence of disasters is posttraumatic stress disorder (PTSD), which includes recurrent intrusive recollections, a heightened threat appraisal, avoidance of reminders of the event, and self-conscious emotions such as guilt and shame (American Psychiatric Association, 2000). Despite a rapidly expanding corpus on traumatic stress, investigations are only beginning to explore the consequences of disasters in the long term.

Extant empirical long-term investigations suggest that chronic full and subsyndromal PTSD, or severe posttraumatic stress, is present in one-fifth to one-fourth of survivors 10–36 years after events such as oil platform disasters (Boe, Holgersen, & Holen, 2011; Hull, Alexander, & Klein, 2002), a devastating flood (Green et al., 1990), a mudslide (Favaro, Zaetta, Colombo, & Santonastaso, 2004), and the present ferry disaster (Arnberg, Eriksson, Hultman, & Lundin, 2011a). Similarly, the prevalence of other anxiety and mood disorders seems to be higher in disaster survivors than in controls decades after the event (Boe et al., 2011).

Cognitive theories of PTSD propose that without active engagement in trauma-related content, traumatized individuals may not experience a significant relief from their symptoms (Brewin & Holmes, 2003; Ehlers & Clark, 2000). Congruent with the theoretical accounts of how PTSD is maintained, social support is important in the recovery process of the victims (Kaniasty & Norris, 2009; Norris et al., 2002). The support from others serves to bolster the individual to confront reminders and gives opportunities for an active elaboration of the distressing event (see Thoits, 2011). Among the risk factors for long-term PTSD, social support is of particular interest as it can be subjected to post-disaster interventions and it may influence the process of adaptation across a long time span. In addition, disasters have unique features that may affect characteristics of support such as media coverage and involvement of governmental and other organizations (Kaniasty & Norris, 2009), which in turn may affect the processes whereby support is associated with distress (see Hobfoll, Dunahoo, & Monnier, 1995).

Long-term disaster studies serve an important role in estimating the psychological burden of disasters, as disaster-related distress may appear or resurface several years after the event (Arnberg, Rydelius, & Lundin, 2011b; Boe, Holgersen, & Holen, 2010). A limitation of present long-term studies, however, is that they have largely constricted their focus to the enumeration of psychological symptoms and disorders. Such a top-down approach may prevent a complete understanding of long-term consequences and multi-method approaches have been called for long since (Lyons, 1991). For example, studies from recent years have pointed to the potential for posttraumatic growth (Helgeson, Reynolds, & Tomich, 2006), a concept that reflects personal and positive consequences attributed to the traumatic experience (Tedeschi & Calhoun, 1996). Studies have shown conflicting findings, however, in that posttraumatic growth has been linked to higher levels of distress (Helgeson et al., 2006; Holgersen, Boe, & Holen, 2010). More exploratory work may be needed in order to understand salient aspects in survivors’ long-term adaptation.

Studies of long-term effects of traumatic events that have employed multi-method approaches highlight the complexity in long-term recovery in survivors from prolonged traumas such as the Holocaust (Suedfeld, Krell, Wiebe, & Steel, 1997) and child sexual abuse (Banyard & Williams, 2007). To this end, mixed-methods designs can serve a valuable purpose (Creswell & Zhang, 2009) as a procedure for collecting and integrating both numerical and narrative data within a single study for the purpose of gaining a better understanding of the phenomenon under study (Tashakkori & Teddlie, 2003).

In 1994, a passenger and car ferry with 186 crew members and 803 passengers on board capsized and sank in the Baltic Sea. Stormy weather conditions hampered effective rescue operations. Many floated for hours in life rafts and boats (water temperature was 10**°**C/50**°**F) before rescue helicopters arrived. There were 137 who survived and 852 people who perished (Joint Accident Investigation Commission of Estonia, Finland, and Sweden, 1997). The primary aim of this study was to examine the long-term psychological consequences in survivors from this disaster using both structured clinical interviews and open-ended data collection. A second aim was to examine what the participants experienced as salient issues with regard to disaster-related social support.

## Methods

### Procedure

In previous studies of this sample, survivors with a Swedish domicile were sent mail surveys at 3 months, 1 year, 3 years, and 14 years after the event and self-reported posttraumatic stress and its association with bereavement and dissociation was assessed (Arnberg et al., 2011a; Eriksson & Lundin, 1996). In the 14-year survey, respondents were asked for consent to personal interviews. Because of the small number of eligible participants, the aim was to include as many as possible of the total sample instead of random or purposive sampling. Those who consented to an interview were invited 10 months later with another letter providing an opportunity to decline participation. The interviewer (F. K. A.) met with the participants, most often in their own home. The interviews took 5 months to complete because of the traveling required due to the fact that the participants lived in all parts of Sweden. The interview commenced with establishing rapport and demographic data collection. Then followed the qualitative data collection, a break, and finally, a structured clinical interview. Hence, the interviewer was not aware of the participants’ diagnostic status during the qualitative data collection. In addition, the interviewer was blind to the participants’ survey responses. The participants provided written informed consent before the interviews. They did not receive any financial reimbursement or compensation.

The interviews were audiotaped and the mean duration was 118 min (range = 85–164 min). The interviews were transcribed verbatim, producing approximately 400 pages of single-spaced text. The transcribers were administrative staff with extensive experience of the task not involved in any other parts of the study. The interviewer checked parts of the transcriptions against the tapes, which did not render any alterations in the transcriptions. Identifying information in the excerpts has been altered to preserve confidentiality. The Regional Ethical Review Board in Uppsala, Sweden approved the study.

### Participants

There were 51 survivors with a Swedish domicile. Eligible participants consisted of 49 survivors who were invited to participate in the survey after 14 years (one was deceased and one was not traceable). Thirty-four (69%) survivors responded to the 14-year survey. Of these, 25 (74%) consented to an interview; however, one later declined to participate and two could not be reached. Thus, the final sample (*n =*22) constituted 45% of the original sample of survivors with a Swedish domicile. A description of survivors who participated and who declined to participate is shown in Table 1.

**Table 1:** Demographics, disaster exposure, and posttraumatic stress in participants and non-participants

	Participants (*n*=22)		Non-participants (*n*=12)	
		
Variable	*N* (%)	Median (range)	*N* (%)	Median (range)
Gender (men)	17 (77)		7 (58)	
University education	12 (55)		5 (42)	
Full-time employment/retired	13/5 (82)		6/2 (67)	
Cohabiting	14 (64)		8 (67)	
Bereaved	5 (23)		5 (42)	
Received therapy^a^	5 (23)		3 (25)	
Age		54 (35–79)		57 (34–75)
IES-R^b^		21 (0–86)		36 (4–82)

*Note*: IES-R, Impact of Event Scale—Revised.

a Psychotherapy and/or pharmacotherapy for severe stress reactions.

b Data from a mail survey approximately 1 year before the interviews.

The final sample was predominantly male and included individuals who identified themselves as healthy and reported single or no episodes of extended sick leave throughout their school years and adult life. The majority of participants was currently employed or had retired from their occupation. Few had been unemployed for any longer period, if at all, in adult years. Two participants were currently unemployed. Although the evacuation was mentally traumatic, none sustained physical injuries that were still an issue today. Several survivors participated in a crisis group that held monthly meetings during the year following the event and partook once or more in annual meetings during the following 10 years.

### Measures and analysis

The Impact of Event Scale—Revised (IES-R; Weiss & Marmar, 1997) is a widely used measure of posttraumatic stress with good psychometric properties. This version of the IES-R assesses the frequency of 22 stress reactions as pertaining to a specific event, yielding a total score of 0–110 (each item is scored 0, 1, 3, or 5) where scores of ≥40 denote significant posttraumatic stress (Sveen et al., 2010). The IES-R was administered in the 14-year survey that was sent out approximately 1 year before the interviews and the total scores were used to compare participants and non-participants.

Open-ended questions in the interview protocol asked about the effect of the disaster on participants’ lives (e.g., “Does the event affect your life today? In what way?”). Two questions asked the participants to describe their experiences of support across the 15 years, first from significant others and then from other sources (“How did you perceive any support from your friends and family/others close to you?”). To maintain similarity across interviews, follow-up questions were restricted to asking the participant to describe or specify the time, duration, and frequency of consequences and support, and the sources of support. The participants were also asked to give examples of their answers when appropriate.

We conducted an inductive thematic analysis at the semantic level (Boyatzis, 1998). This method was appropriate because it is aimed at extracting common themes in data but not at the building theory, which we felt was premature at this point, and it focused on the statements of the participant rather than on the coders’ interpretation, which facilitates comparisons with other studies of survivors’ experiences.

F. K. A. reviewed the corpus for items relevant to long-term consequences and social support with an inclusive approach, yielding two data sets; where appropriate, items were included in both sets. One item could consist of an utterance or one or more consecutive sentences. F. K. A. then generated provisional themes from the data that were revised in a recursive process according to gathering rich descriptions of the cases while keeping consistency within and distinctiveness across themes. During this iterative process, data verification procedures also included reviewing and resolving disconfirming evidence, and academic adviser's auditing. After saturation of the data, T. L. performed an independent coding of the social support segments of the transcriptions. Any discrepancies were discussed and resolved by going back to the iterative process. NVivo Software v.7 (QSR International) was used for data storage, coding, and theme development.

The structured clinical interview for the Diagnostic and Statistical Manual, 4th edition, Axis I disorders (SCID-I; First, Spitzer, Gibbon, & Williams, 1997/1998) was used to assess current and lifetime disorders. Owing to time constraints, the modules included in this study were axis I disorders associated with traumatic experiences (see Norris et al., 2002): anxiety disorders, mood disorders, and alcohol and substance abuse. In the PTSD module, the participants were also asked to describe their experiences during the event and whether they were bereaved in the event. They were classified as bereaved if a significant other (i.e., family member, close friend) died in the event. The Life Events Checklist (Gray, Litz, Hsu, & Lombardo, 2004) was used to supplement the SCID when screening for lifetime exposure to traumatic events. The checklist consists of 16 items inquiring about the experience of 16 different potentially traumatic events and has adequate psychometric properties, such as temporal stability and convergent validity (Gray et al., 2004). PTSD was classified as subsyndromal if at least one avoidance symptom was fulfilled and all other criteria were met, which is in accordance with the World Health Organization's (1993) classification of PTSD (see Norris & Slone, 2007; Stein, Walker, Hazen, & Forde, 1997). The interviewer was a male licensed psychologist with additional training specific to SCID and with experience of using SCID in both research and clinical settings. Demographic data were collected by closed and open-ended questions at the start of the interview.

The proportions of cases are presented with 95% confidence intervals (CIs) and were calculated using SPSS Statistics v.20 (IBM, Chicago, IL). To highlight any contrasts between the clinical interview and the self-reported consequences, we assessed whether PTSD was related to the presence of salient positive consequences and barriers to support in participants’ narratives. Because of the association between PTSD and depression and bereavement (Norris & Slone, 2007), we also assessed whether depression and bereavement were related to these themes. Fisher's exact test was used with significance set at *p*<0.05 (two-tailed).

## Results

### Long-term consequences

#### Psychiatric caseness

Ten participants had experienced one or two additional events that fulfilled the A-criterion of the DSM-IV diagnosis of PTSD and one participant had experienced five events. Twenty participants indicated that the ferry disaster was the worst event they had experienced. No case of pre-disaster PTSD was identified and all cases of PTSD were associated with the ferry disaster. The lifetime prevalence thus equated the post-disaster incidence, which was 45% (10/22), 95% CI [25.9, 66.2%] for full and subsyndromal PTSD (Table 2). All cases with early onset reported an endpoint of the PTSD symptoms or functional impairment 2–24 months after the event (*m*=14 months). Cases with current PTSD recalled a late onset or a relapse after a period of time when the symptoms were not functionally impairing. Regarding symptom clusters, intrusions were prevalent whereas avoidance and numbing symptoms were less prevalent and were, effectively, the threshold criterion for receiving a diagnosis of lifetime PTSD. All cases of situational phobias were related to travel (i.e., fear of travel by air and sea). A history of social phobia that predated the disaster was observed in two participants. The lifetime prevalence of any anxiety disorder (excluding subsyndromal PTSD) was 36% (8/22), 95% CI 18–55%.

**Table 2:** Prevalence of anxiety, mood and substance use disorders and posttraumatic symptom criteria in 22 survivors 15 years after the disaster

	Current		Post-disaster onset		Pre-disaster onset	
			
Disorder	*n*	% [95% CI]	*n*	% [95% CI]	*n*	% [95% CI]
PTSD	1	4.5 [0.3, 18.5]	7^a^	31.8 [15.1, 52.5]	0	
Subsyndromal PTSD^b^	2	9.1 [1.6, 25.5]	3	13.6 [3.6, 31.7]	0	
Intrusion	13	59.1 [38.4, 77.8]	21	95.5 [81.5, 99.7]		
Avoidance	3	13.6 [3.6, 31.7]	7	31.8 [15.1, 52.5]		
Hyperarousal	8	36.4 [18.6, 57.2]	13	59.1 [38.1, 77.9]		
Functional impairment	3	13.6 [3.6, 31.7]	10	45.5 [26.0, 65.9]		
Specific phobia	3	13.6 [3.6, 31.7]	3	13.6 [3.6, 31.7]	0	
Social phobia	0		0		2	9.1 [1.6, 25.5]
Major depression	3^c^	13.6 [3.6, 31.7]	7	31.8 [15.1, 52.5]	1	4.5 [0.3, 18.5]
Alcohol abuse	0		3	13.6 [3.6, 31.7]	1	4.5 [0.3, 18.5]
Any disorder^d^	5	22.7 [8.8, 42.6]	12	54.5 [34.1, 74.0]	4	18.2 [6.0, 37.3]

*Note*: Disorders were assessed after 15 years by the Structured Clinical Interview for DSM-IV. Anxiety, mood, and substance use disorders not found in the sample are omitted from the table. PTSD = posttraumatic stress disorder.

a All participants reported the ferry disaster as their index trauma.

b At least one avoidance symptom was fulfilled and all other criteria were met.

c Current PTSD symptoms were highly distressing for two cases with recurrent episodes.

d Does not include subsyndromal PTSD. The total *n* does not correspond to the *n* for all disorders because of comorbidity.

Among mood disorders, only cases of major depression were seen. Six cases reported one or two episodes and two reported recurrent episodes. Current PTSD symptoms were prominent in the cases with recurrent depression. Participants with a lifetime diagnosis of full or subsyndromal PTSD (*n*=10) had a 50% probability of having a lifetime major depressive episode, whereas the probability was 25% for those without PTSD. Previous alcohol abuse was noted in four participants. Two cases reported using alcohol to cope with posttraumatic stress; and one case reported that other life circumstances after the disaster was the cause of the alcohol abuse. The three cases with post-disaster onset had developed full PTSD before onset of alcohol abuse. No cases of generalized anxiety disorder, panic disorder, or drug abuse or dependency were seen. The cases with a pre-disaster disorder also developed a disorder after the disaster. Thus the lifetime prevalence of having any disorder equated the post-disaster incidence (Table 2).

In the thematic analysis of long-term consequences, we found 25 categories (covering 87% of the data in this set). The main themes were, in order of decreasing salience in the narratives: (1) character change, (2) survivor identity, and (3) emotions. Below, the number of participants relating to each category is presented in square brackets.

#### Character change

Many participants felt that in the long term they had changed in areas of personal growth [12] and existential awareness [12]. Personal growth was expressed as a greater confidence in one's ability to manage stressful situations [6]: “I trust myself more…. I can handle diverse situations, which is assuring if things happen that make others shiver [with fear]”. The greater confidence in oneself was related to a shift in life values: for example, that the opinions of others were less important [4]. Existential awareness was reflected in the participants’ sense of “living in the present” to a greater extent, as well as more “life-affirming” and, “grateful toward life”, and not being “bothered by petty things.”

Linked to both areas of character change were positive appraisals of having developed a more sensitive risk assessment noted in a few participants [3]: “Be prepared! Anything can happen. It's positive, I mean, if you watch your footstep you're smart.” Being more apprehensive, however, was also framed negatively [2], being associated with situational fears: Some participants disclosed situational fears [7] related to travel by air or sea, or stormy weather, for example, “I've become more cowardly about certain things. I mean, this with me travelling by airplane.”

The areas of character change were contrasted against PTSD and, for reference, post-disaster depression and traumatic bereavement. As seen in Table 3, participants who fulfilled criteria for PTSD at some point after the event were more likely to express personal growth whereas neither depression nor bereavement were seemingly related to the salient aspects of character change.

**Table 3:** Associations between positive consequences, barriers to social support and traumatic bereavement, post-disaster PTSD and depression in 22 survivors 15 years after a disaster

	Character change					Barriers to support			
			
	Total	Personal growth (*n*=12)		Existential awareness (*n*=12)		Others’ affliction (*n*=10)		Experiential dissimilarity (*n*=7)	
				
Predictor	*n*	*n* (%)	χ^2^	*n* (%)	χ^2^	*n* (%)	χ^2^	*n* (%)	χ^2^
PTSD									
Yes^a^	10	8 (80)	4.79*	6 (60)	0.22	4 (40)	0.22	3 (30)	0.03
No	12	4 (33)		6 (50)		6 (50)		4 (33)	
Depression									
Yes^a^	7	4 (57)	0.03	4 (57)	0.03	3 (43)	0.03	1 (14)	1.46
No	15	8 (53)		8 (53)		7 (47)		6 (40)	
Bereaved									
Yes	5	2 (40)	0.55	3 (60)	0.78	3 (60)	0.55	0 (0)	3.02
No	17	10 (59)		9 (53)		7 (41)		7 (41)	

*Note*: Numbers for narrative data represent cases whose narratives included the relevant theme. Fisher's exact test was used to assess whether diagnostic or bereavement status was related to the presence of themes found in the qualitative analysis. All *df*=1.

a Only cases with post-disaster onset were included (all cases of PTSD and all but one case of depression).

* *p*<0.05

#### Survivor identity

A struggle with being ascribed a survivor identity was observed in both bereaved [2] and non-bereaved participants [6]. An excerpt offers a summarizing description: “people around me sometimes see me more as someone who was aboard the ferry than as me…. I carry a little bit of other people's fears and I am seen as someone who can handle difficult situations.” Being attributed a survivor identity was regarded as negative. Several of the participants even had decided to no longer disclose that they were survivors [7], which was associated with varying degrees of reservation when meeting new people: “I won't let acquaintances close to me…. I keep a guard up, kind of, against outsiders. I don't see it as obvious that they have the right to know me.” The participants had also made use of the survivor identity, such as when trying to help and advise others through disclosure of their experiences from the event [5] (“turning my bad experience into something good for others”). Although they acknowledged that others assigned to them positive characteristics as survivors, expressing positive effects themselves of such a disastrous event was not without difficulties. Either they stated that they were reluctant to, or did not disclose such effects [4], or they supplemented their description of any positive effects with statements of the horrific nature of the event [3].

#### Emotions

The event was still an emotional experience for some of the participants [9]. Feelings of sadness and sorrow, and irritation and frustration were found in these participants’ narratives. In essence, emotional content seemed to be related to prolonged consequences that the participant could not control. Frustration and irritation were related to the still ongoing disputes about the cause of the disaster: “It's been shuffled back and forth all the time, and is always coming back – 5 years, 10 years after.” Sadness and sorrow were related to bereavement but also pondering over their fate, “if this hadn't happened…” and to the repercussions of the event on their lives. Only one participant explicitly reported consequences mapping onto posttraumatic stress (i.e., having recurrent nightmares). Yet, eight participants acknowledged toward the end of the interview (after the SCID) that they anticipated nightmares or intrusive thoughts over the following day or days.

### Social support

The majority of participants remarked that there had been, and still was, sufficient availability of support relative to their needs [19]. They stated that their need for support declined sharply during the first year and leveled approximately 5 years after the event. One male participant, however, expressed an increasing need for support, reportedly because of a slowly declining reluctance on his part to disclose emotional information. Several participants described an increased need of support in 2004, which they explained was due to the 10-year commemoration of the event and to the 2004 Indian Ocean tsunami.

In the thematic analysis, we found that except for the need for support, the participants’ descriptions mainly involved two related themes: sources of support and barriers to support. In nearly all narratives [20], the participants described themselves as the key agents in their recovery, for instance, in the assessment of others’ capacity for offering support. We failed to detect statements implying that the participants were merely passive recipients of support.

#### Sources of support

The family was a common source of social support [16], yet the participants had concerns about seeking their support. For six participants, it was because they felt that their significant others had at some point indicated that they ought to move on in life, either explicitly (“are you not well yet?”) or implicitly (“it's so long ago now”).

Some participants wanted a professional therapeutic context for disclosure of event details [6], noted both in participants with a previous therapeutic contact (“there were things I did not want to expose them [my family] to”) and in participants without previous contact (“If I would need to talk about it, I would have sought out a professional to talk to”). Others had found support in their colleagues: “in some way they [my colleagues] could provide an aid that my own family couldn't really give.” This excerpt continues with an example of why seeking support from the family was difficult for many: “Because they [my colleagues] could care, without caring too much.”

#### Barriers to support

Many participants gave detailed descriptions of why they did not seek or receive support from significant others [16]. We found two main reasons, the affliction of others and experiential dissimilarity, which seemed to be related to whether the participant's family had suffered a loss (Table 3). For bereaved participants, “one's own family may also be a part of the trauma,” and the affliction of significant others was a reason not to seek their support: “[I] really rather just wanted to die. I thought, why didn't I die too, but I couldn't say that to those who were so happy that I was still alive.” The distress in others could lead to reversed roles in the support transactions, which was associated with frustration, disappointment, or loneliness: “Eventually, I understood that it was not me that they wanted to help. They wanted to help themselves. When you come to that insight, you get disappointed, really disappointed.”

Although participants without family bereavement also noted others’ affliction [7], they seemed to frame the barrier as mainly due to an experiential dissimilarity [7], pointing to the different experiences between themselves and family members. One excerpt summarizes the descriptions of several participants:

[My family] had a very traumatic day—they didn't know if I was dead or alive. … I think that it hit them harder than it hit me. … I was right in the middle of the trouble … but the others [family members] had in fact a very traumatic experience.

Concerning the survivor meetings during the first year, one participant expressed “We who were there [the survivors] understood what we had been through, and that was very important because no one else could understand what we had been through.” Apart from experiential dissimilarity and the adversity of others, five participants briefly noted that they thought that if they talked any more about the event, their family and friends would see them as “being stuck in the event.”

## Discussion

This study described the psychological consequences of a disaster and detailed the survivors’ perceptions of social support in a long-term perspective. Twelve of the 22 participants had experienced anxiety disorders, depression, or substance abuse with a post-disaster onset. Full and subsyndromal PTSD accounted for 10 cases and subsided mainly within the first 2 years after the disaster. In contrast, the central long-term consequences as construed by the survivors were related to character change, survivor identity, and negative emotions. The need for social support persisted for several years, although there was adequate availability. Barriers to seeking support were frequently mentioned, the main reasons being the affliction of significant others and experiential dissimilarity.

In previous investigations of disasters the prevalence rates of full or subsyndromal PTSD, or severe posttraumatic stress, were one-fourth to one-fifth of survivors 10–36 years after the event (Boe et al., 2011; Favaro et al., 2004; Green et al., 1990; Hull et al., 2002). About half of the survivors from these events developed PTSD at some point after the event, which corresponds well with the present findings, whereas the current prevalence of PTSD was somewhat lower in this study. The lower rate may be related to event characteristics in that this event did not affect the survivors’ ability to function at work, in the way an oil platform disaster would. The small sample and risk of selection bias in this study, seen also among other long-term follow-ups of disasters, limit the reliability of findings from individual studies and comparisons among them. Nevertheless, there seems to be high concordance across studies regarding the occurrence of other anxiety disorders, mood disorders, and substance use disorders.

Few participants fulfilled the avoidance criterion, which thus served as a threshold criterion for receiving a diagnosis of PTSD. This finding has been noted in studies after other high-profile events (Heir, Sandvik, & Weisæth, 2009; North et al., 1999). Such events presumably are associated with more inquires about the survivor's experiences, or less stigma (see Ehlers & Clark, 2000), which may offset inclinations to avoid trauma-related stimuli. Moreover, the majority of participants fulfilled the intrusion criterion, which may reflect a normative rather than abnormal long-term response to disasters (Norris & Wind, 2009).

Indeed, despite the high prevalence of one or more PTSD symptoms we found few spontaneous reports of posttraumatic stress and descriptions of emotions were given quite modest attention by the participants. Interestingly, this discrepancy illustrates that many participants seemed to construe the consequences affecting broadly their character and relations. The absence of psychiatric consequences in the narratives may also reflect that symptoms grow less salient with time. However, the participants may downplay the disclosure of emotional content in favor of structuring it around problem solving, as suggested in a content analysis of interviews with Holocaust survivors (Suedfeld et al., 1997). Again, this could be influenced by the gender of the participants and the interviewer. However, also noted by Suedfeld et al. (1997), this pattern of presentation may be congruent with the actual accomplishments of the interviewees in that they managed to survive the event and then to overcome difficulties in its aftermath. Regardless of the causes of this discrepancy, studies that rely on a bottom-up approach carry a risk of failing to account for important adverse consequences, whereas studies that rely on a top-down approach may fail to adequately describe the breadth of the long-term consequences and may overstate the influence of individual symptoms on participants’ daily lives.

The positive consequences found in the thematic analysis are well described by aspects of posttraumatic growth (Tedeschi & Calhoun, 1996): Personal strength and appreciation of life were prominent in the narratives. However, other features of posttraumatic growth were not found to any greater extent (e.g., more intimate relationships), or were not found at all (e.g., strengthened spiritual beliefs). The scarcity of these features of growth may be related to the sample being predominantly male in that gender differences in posttraumatic growth seem to be more pronounced for these two aspects (men perceive less growth than women; see Tedeschi & Calhoun, 1996). Further adding to the literature on the complexity of growth after trauma (Helgeson et al., 2006), the participants without a history of PTSD were less likely to express personal growth than were participants with a history of PTSD, whereas no such tendencies could be observed related to depression or bereavement. Bereavement was associated with worse long-term outcome in the 14-year survey of the survivors from this incident (Arnberg et al., 2011a) and depression is typically influenced by ongoing stressors rather than directly by the event (Tracy, Norris, & Galea, 2011). One may speculate that depression and bereavement hinder a transformation from adversity to growth by being tied more to the present compared to PTSD that is directly linked to the event.

The survivors were hindered in seeking support mainly by experiential dissimilarity and the affliction of their significant others. Consistent with reports from other disasters, the majority of participants noted that there was ample availability of support (Kaniasty & Norris, 2009). The long-term need may have been biased upwards by episodes of an increased need for support, such as during the 2004 tsunami, which had a particularly large impact on the country (Bergh Johannesson et al., 2009). Also, these findings lend support to Thoits’ (2011) suggestion in that the stress experience, rather than demographics and intimacy (Cohen & McKay, 1984), was central to expressing experiential similarity, and experiential dissimilarity appeared to be a more subtle barrier when bereavement was involved. Because of the retrospective nature of the data these findings should be regarded as preliminary. Nevertheless, these findings highlight areas important to post-disaster psychosocial interventions (Brymer et al., 2006), such as the prolonged need of support.

There are some limitations to this study. First, we aimed to include all survivors rather than to attempt purposive sampling. We are cognizant of the limitations of a small sample size and non-random recruitment of participants. There were indications of a selection bias toward non-bereaved participants and participants with less posttraumatic stress. However, our sample included a fair coverage of different experiences and posttraumatic stress levels, which strengthens the thematic analysis. Finally, since a single person conducted all interviews we tried to minimize experimenter bias by investigator triangulation. Nonetheless, the fact that both the interviewer and most participants were male may have influenced the participants’ reporting and limit the generalizability of the findings.

The main strength of this study was that the inclusion of open-form queries made available a complete representation of long-term consequences, contrasting data emerging from simply enumerating cases with psychiatric morbidity with issues pertinent to survivors. Further studies with a broader scope may contribute to a more comprehensive description of long-term consequences (Creswell & Zhang, 2009).

In conclusion, this study corroborates previous findings that a single, extreme event can lead to long-term adverse mental health consequences in a minority of survivors. These findings offer a complementing picture through the survivors’ accounts, which emphasized both positive and negative consequences and were related not to symptom presentation but to changes in their view of themselves and in how others view them. This study notes that social provisions from both significant and similar others may be important long after the psychological suffering associated with the first years post-disaster has subsided. It is hoped that these findings can further long-term investigations after disasters, ultimately to the benefit of survivors who struggle with posttraumatic distress for decades.
